# Engineering Bone-Implant Materials

**DOI:** 10.3390/bioengineering6020051

**Published:** 2019-06-09

**Authors:** Mohammad Elahinia, Hamdy Ibrahim, Mohammad Javad Mahtabi, Reza Mehrabi

**Affiliations:** 1 Dynamic and Smart Systems Laboratory, Department of Mechanical Industrial and Manufacturing Engineering, University of Toledo, Toledo, OH 43606, USA; Reza.mehrabinejad@gmail.com; 2 College of Engineering, University of Tennessee at Chattanooga, 615 McCallie Ave, Chattanooga, TN 37403, USA; hamdy.m.elsayed@gmail.com (H.I.); mjmahtabi@gmail.com (M.J.M.)

This special issue is dedicated to the simulation as well as experimental studies of biomechanical behavior of biomaterials, especially those that are used for bone implant applications. It is worth noting that orthopedic trauma (fractures) alone represented one-half of all the disease or injury-related hospitalizations in 2004–2005 [1]. Individualized care and better outcome require enhanced functional biomaterials. For device design and customization, precise modeling of the biomechanical behavior of the resulting biomedical devices is essential. These models are at the heart of the surgical planning methodologies towards more personalized medicine with patient-specific implants. In one review and three research articles, this special issue covers a range of computational, analytical, and experimental studies related to bone implant-materials and bone reconstructive surgeries.

In the research article written by Mebarki et al. [2], a solution to the problem of bone graft regeneration in the case of prosthesis revision was proposed. This approach was designed and implemented for the case of revision of the implanted inverted shoulder prosthesis using the bony increased offset–reversed shoulder arthroplasty (BIO–RSA) method. The shoulder joint was reconstructed in 3D using Mimics and RapidForm and the prosthesis was designed using SolidWorks. Using finite element analysis, the behavior of different types of biomaterials that can replace bone grafting was studied for three abduction motions of the arm in three positions (30°, 60°, and 90°). The biomaterials investigated in this study were titanium, stainless steel, poly methacrylate (PMMA), and ultra-high molecular weight polyethylene (UHMWPE). This study showed that the use of graft made of UHMWPE is expected to result in minimized stresses and more protection to the prosthesis from the risk of fatigue failure due to the close mechanical response of UHMWPE to that of bone.

The effect of varying levels of medial collateral ligament deficiency on elbow joint stability was studied by Rahman et al. [3] using subject-specific computational models. The studied ligament conditions are (i) all intact ligaments, (ii) isolated medial collateral ligament (MCL) anterior bundle deficiency, (iii) isolated MCL posterior bundle deficiency, and (iv) complete MCL deficiency. The models considered multiple bone geometries and discretized cartilage representation. The results of this study showed that the use of the anterior bundle has the biggest influence on joint kinematics and contact characteristics.

During the last decade, the use of bioresorbable metals in biomedical applications (e.g., Mg, Fe and Zn alloys) has been an area of considerable interest, with numerous investigations working towards the development of biodegradable bone fixation hardware and stents. Amerinatanzi et al. [4] developed a model for predicting the degradation behavior of a biocompatible Mg–Zn–Ca alloy in vitro using a customized FORTRAN user material subroutine (VUMAT) and the finite element (FE) solver Abaqus/Explicit. This was performed using a continuum damage mechanism (CDM) FE-based model. The development of models increases the ability to design biodegradable Mg alloys for various biomedical applications.

The last article in the special issue, written by Nematollahi et al. [5], provides a comprehensive summary on the biomedical applications of NiTi shape memory alloys (SMAs) in assistive and rehabilitation devices. The use of SMAs as functional devices implanted in the human body is one of the well-established and progressing areas in the literature. Such functional devices, in the form of bone implants, stents, filters, and actuators, are possible due to several properties of SMAs, such as their shape memory behavior, superelasticity, and biocompatibility. The review presents the state of the art of the application of SMAs in actuator form, where feedback control enables innovative medical devices. In addition, the two major subsets of these devices, prosthesis and orthosis, are reviewed. Finally, the paper identifies several possible future directions of SMA-related research in the area of assistive and rehabilitation devices.

The guest editing team of this special issue would like to thank the authors as well as the staff of *Bioengineering* for their valuable contributions.
